# Painless Aortic Syndrome in a Patient with Syncope and Globus Sensation: A Case Report

**DOI:** 10.5811/cpcem.20348

**Published:** 2024-11-09

**Authors:** Gavriel Rosenfeld-Barnhard, Jessica R. Jackson, Kendra A. Mendez, Kraftin E. Schreyer

**Affiliations:** Temple University Hospital, Department of Emergency Medicine, Philadelphia, Pennsylvania

**Keywords:** aortic dissection, globus sensation, case report

## Abstract

**Introduction:**

Aortic dissection is a devastating clinical entity with a variety of presentations and requires prompt recognition and management. To our knowledge this is the first reported case of a patient who presented with a globus sensation and was diagnosed with an aortic dissection prior to clinical deterioration.

**Case Report:**

The patient presented with an episode of near-syncope and globus sensation. Imaging studies revealed a type A aortic dissection with hemopericardium requiring emergent operative intervention. Unfortunately, the patient’s course was complicated by significant hemorrhage and periods of hypotension, and the family ultimately decided to pursue comfort care.

**Conclusion:**

Aortic dissections can present with diverse and elusive symptoms, which can mimic other more common conditions, potentially leading to misdiagnosis and delayed intervention

## INTRODUCTION

Acute aortic dissection is one of the most devastating cardiovascular pathologies encountered in the emergency department (ED), with high levels of morbidity and mortality. Due to the potential rapid clinical deterioration, prompt recognition of this condition and appropriate timely diagnostic evaluation and treatment are crucial. The widely taught clinical presentation of aortic dissection is sharp, tearing chest pain that radiates to the back. In clinical practice, however, there is a wide range of signs and symptoms of aortic dissection including syncope, hypotension, and focal neurologic deficits. We describe an uncommon presentation of a type A aortic dissection in which the patient’s primary clinical symptom on initial presentation to the ED was a globus sensation.

## CASE REPORT

A 60-year-old male presented to the ED after an episode of near-syncope associated with diaphoresis while walking to church, with emergency medical services reporting low blood pressure at the scene. On arrival, he endorsed a tight sensation in his throat but otherwise denied chest pain, shortness of breath, back pain, nausea, or vomiting. He had a medical history of hyperlipidemia, well-controlled hypertension on amlodipine and remote history of prostate cancer status post radical prostatectomy but was otherwise unremarkable. He had a remote smoking history of 10 pack-years but otherwise no drug use.

On examination, the patient was alert and oriented and in no distress. Initial vital signs were temperature 36.7° Celsius, heart rate 72 beats per minute, blood pressure 105/72 millimeters of mercury (mm Hg) on the left arm, and respiratory rate 16 breaths per minute with an oxygen saturation of 98% on room air. Initial examination was notable for regular rate and rhythm with a normal S1 and S2, lungs were clear to auscultation bilaterally, and his abdomen was benign. Radial and dorsalis pedis pulses were 2+ bilaterally. The neurologic exam was normal with symmetric strength in his bilateral upper and lower extremities without sensory deficits. The neck exam was notable for a midline trachea, no palpable masses. He also had an unremarkable oropharyngeal exam.

**Image 1:**
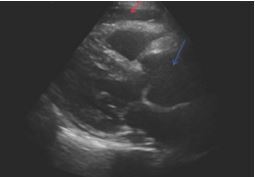
Point-of-care echocardiogram in parasternal long view demonstrating pericardial effusion (red arrow) and a dilated aortic root (blue arrow).

Diagnostic workup was notable for an initial electrocardiogram that was normal sinus rhythm with a normal rate, normal intervals, and no evidence of ischemic changes or arrythmias. A chest radiograph showed a mildly enlarged heart size and abnormal contour of the thoracic aorta. While awaiting laboratory studies, point-of-care ultrasound showed an enlarged left ventricular outflow tract (LVOT) measuring 4.4 centimeters (cm) (reference range: 1.6–2.4 cm)and evidence of a pericardial effusion without evidence of tamponade (Image 1).

**Image 2:**
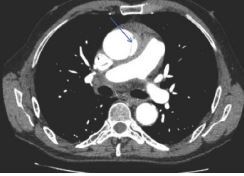
Computed tomographic angiography of the chest showing a dissection flap (arrow) of the proximal aorta with intramural hematoma.

These ultrasound findings were highly suspicious for acute aortic pathology and prompted an emergent computed tomography angiography (CTA) of the chest, abdomen, and pelvis, which showed an intramural hematoma of the aortic root extending along the right pulmonary arterial wall and along the aorta to the aortic arch; dilation of the ascending aorta with a dissection flap with associated inflammatory changes of the mediastinum; and aneurysmal dilation of the infrarenal abdominal aorta, measuring 3.1 cm in the midportion and 3.4 cm just proximal to the bifurcation (Image 2).

While imaging was being obtained, blood tests began to result, which were notable for a white blood cell count of 13.7 × 10^3^ per cubic millimeter (K/mm^3^) (reference range: 4.0–11.0 K/mm^3^), hemoglobin 14.2 grams per deciliter (g/dL) (14.0–17.5 g/dL), platelets 328 K/mm^3^ (150–450 K/mm^3^), creatinine 1.44 milligrams (mg)/dL (mg/dL) (0.80–1.30 mg/dL) (last from one year prior 1.01 mg/dL), and high sensitivity troponin I 12 nanograms per liter (ng/L) (12–76 ng/L [males]). With concern for an acute aortic syndrome after the initial ultrasound was performed, a D-dimer was later obtained, which was elevated at 1,846 ng/mL (0–500 ng/mL). Otherwise, all other labs were within normal limits.

**CPC-EM Capsule**

What do we already know about this clinical entity?

*Aortic dissection is a life-threatening condition with diverse symptoms, often requiring quick recognition and management to prevent deterioration.*

What makes this presentation of disease reportable?

*This is the first reported case of an acute aortic syndrome presenting with painless globus sensation, diagnosed before clinical deterioration.*

What is the major learning point?

*Atypical presentations like globus sensation in aortic dissection require high suspicion, as early diagnosis and management are crucial to improving outcomes.*

How might this improve emergency medicine practice?

*Clinicians must be vigilant in recognizing non-textbook presentations of critical conditions to improve early detection and treatment.*

Given the CTA findings, the cardiothoracic surgery team was emergently consulted with concern for type A aortic dissection. While awaiting surgical recommendations, the patient subsequently developed sudden onset lower back pain associated with nausea, vomiting, and diaphoresis. At this time the patient was noted to have discrepant blood pressures. A right-sided radial arterial line was placed that showed a blood pressure of 64/58 mm Hg, and a left arm automated blood pressure cuff read 154/105 mm Hg. The patient was given 4 mg intravenous (IV) ondansetron, 6 mg of IV morphine, and 800 micrograms (μg) of sublingual nitroglycerine. The patient was subsequently started on esmolol and nitroprusside at an initial rate of 50 μg per kilogram per minute (kg/min) and 0.5 μg/kg/min, respectively, for blood pressure and heart rate management. The patient then reported loss of sensation in his right lower extremity. Re-examination revealed total loss of motor function and no palpable femoral pulses.

A repeat point-of-care ultrasound showed an abdominal aortic flap concerning for propagation of the aortic dissection. The patient was taken emergently to the operating room and, intraoperatively, was noted to have a dissection extending 4 cm above the valve and coronaries. A transesophageal echocardiogram was also performed that showed a tricuspid aortic valve and redemonstrated the dissection extending from the ascending through descending aorta. After a prolonged surgery complicated by hemorrhagic shock, the family opted for comfort care measures on the following day.

## DISCUSSION

An aortic dissection refers to the development of a focal tear in the intimal layer of the aortic wall that results in extension of the tear compromising blood flow to vital organs. The Stanford classification is a well-established system of describing aortic dissections: type A dissection refers to dissections that involve any part of the aorta proximal to the origin of the left subclavian artery and which may also include the descending aorta, while a type B dissection refers to dissections involving the descending aorta or the arch (distal to the left subclavian artery), without the involvement of the ascending aorta. Both entities are relatively rare diagnoses with an annual incidence of approximately 3.5 per 100,000 patients.^1^ Because of the significant morbidity and mortality associated with this condition, a high degree of clinical suspicion is required to make the diagnosis. Large systematic reviews have shown that over 80% of patients found to have an aortic dissection present with complaints either of chest or back pain, and about 10% are painless or may present with symptoms secondary to complications of the dissection.^2^,^3^ The International Registry of Acute Aortic Dissections reports that syncope was present in nearly 20% of type A aortic dissections and is often associated with a severe complication such as cardiac tamponade.^4^,^5^ Due to the difficulty of diagnosis, emergency physicians only suspected aortic dissection in 43% of confirmed cases.^6^

There are only a few case reports describing throat tightness as the initial presentation of patients found to have aortic dissections. In a 2004 case report by Liu and Ng, a patient presented to the ED with acute-onset throat pain and was subsequently discharged after symptomatic management. The patient returned to the ED later that day with worsening sore throat, severe chest pain, diaphoresis, and a syncopal episode and was found to have a type A aortic dissection.^7^ Another case report by Cates et al describes a 58-year-old patient who initially presented with burning throat pain that eventually progressed to chest pain and right lower extremity cramping. The change in the patient’s symptoms prompted a CTA, which revealed the diagnosis of a type A aortic dissection. 8

There is also a phenomenon in the literature called “dysphagia aortica,” which is defined as difficulty in swallowing caused by extrinsic compression of the esophagus due to an ectatic, tortuous, or aneurysmatic atherosclerotic thoracic aorta.^9^ This rare clinical entity is mostly described in case reports and small case studies. Most of the cases are descriptions of patients with weeks to months of dysphagia who, in the process of extensive outpatient workups, are found to have large thoracic aortic aneurysms.^10^,^11^ Although the pathophysiology is not well understood, some case reports suggest external esophageal compression from the aneurysm as the potential cause of the dysphagia. Dilated aortic aneurysms can compress the esophagus, recurrent laryngeal nerve, or superior cervical sympathetic ganglion that in turn can cause dysphagia, hoarseness, or Horner syndrome.^12^ Our case is unique in that the patient acutely developed symptoms suggestive of extrinsic compression of mediastinal structures related to his dissection and dilated aorta.

Our case underscores the critical need for emergency physicians to remain vigilant and open to atypical clinical presentations of aortic dissection. Aortic dissections can present with diverse and elusive symptoms, which can mimic other, more common conditions, potentially leading to misdiagnosis or delayed intervention. In our case as well as has been described in prior case reports, although a sore throat was the initial presenting symptom, advanced imaging diagnosing an aortic dissection was not obtained until after the patient’s clinical deterioration or development of other symptoms. To our knowledge this is the first reported case of a patient who presented with a globus sensation and was diagnosed with an aortic dissection prior to clinical deterioration. This case emphasizes the vital role of clinical acumen and the importance of maintaining a high index of suspicion, even when faced with non-textbook presentations. While the point-of-care echocardiogram was key to obtaining the diagnostic CTA, recognizing the subtle, unconventional signs of aortic dissection is paramount to ensure early diagnosis and timely management, which can significantly impact patient outcomes and save lives.

## CONCLUSION

Due to the significant morbidity and mortality of aortic dissections, particularly of type A dissections, prompt recognition and management is essential. It is crucial to recognize the wide range of initial patient presentations to ensure early identification of this emergent pathology. This case underscores the importance of maintaining a high index of suspicion for aortic dissections and abnormal ultrasound findings, particularly in patients with known risk factors and non-specific symptom presentations that do not fit the “textbook” presentation.
